# The survival outcomes of localized low‐risk prostate cancer, a population‐based study using NCDB

**DOI:** 10.1002/cam4.70060

**Published:** 2024-08-09

**Authors:** Shifeng Mao, Arash Samiei, Yue Yin, Rodney E. Wegner, Angela Sanguino, John Lyne, Ralph Miller, Jeffrey Cohen

**Affiliations:** ^1^ Allegheny Health Network Cancer Institute Pittsburgh Pennsylvania USA; ^2^ Division of Urology Allegheny Health Network Pittsburgh Pennsylvania USA; ^3^ Allegheny‐Singer Research Institute Allegheny Health Network Pittsburgh Pennsylvania USA; ^4^ Division of Radiation Oncology Allegheny Health Network Cancer Institute Pittsburgh Pennsylvania USA; ^5^ Department of Pathology Allegheny Health Network Pittsburgh Pennsylvania USA

**Keywords:** active surveillance, external beam radiation, low‐risk prostate cancer, prostate seed implantation, prostatectomy

## Abstract

**Background:**

The optimal treatment approach for low‐risk prostate cancer (LRPC) remains controversial. While active surveillance is an increasingly popular option, definitive local treatments, including radical prostatectomy (RP), external beam radiotherapy (EBRT), and prostate seed implantation (PSI), are also commonly used. This study aimed to evaluate the survival outcomes of patients with LRPC using a large patient population from the National Cancer Database (NCDB).

**Methods:**

We analyzed data from 195,452 patients diagnosed with LRPC between 2004 and 2015 using the NCDB. Patients were classified based on their treatment modalities, including RP, EBRT, PSI, or no local treatment (NLT). Only patients with Charlson–Deyo comorbidity scores of 0 or 1 were included to ensure comparability. Propensity score analysis was used to balance the treatment groups, and the accelerated failure time model was used to analyze the survival rates of the treatment groups.

**Results:**

After a median follow‐up of 70.8 months, 24,545 deaths occurred, resulting in an all‐cause mortality rate of 13%. RP demonstrated a survival benefit compared with NLT, particularly in patients younger than 74 years of age. In contrast, radiation treatments (EBRT and PSI) did not improve survival in the younger age groups, except for patients older than 70 years for EBRT and older than 65 years for PSI. Notably, EBRT in patients younger than 65 years was associated with inferior outcomes.

**Conclusion:**

This study highlights the differences in survival outcomes among LRPC treatment modalities. RP was associated with improved survival compared to NLT, especially in younger patients. In contrast, EBRT and PSI showed survival benefits primarily in the older age groups. NLT is a reasonable choice, particularly in younger patients when RP is not chosen. These findings emphasize the importance of individualized treatment decisions for LRPC management.

## Graphical abstract

Overall survival of patients with low‐risk prostate cancer.

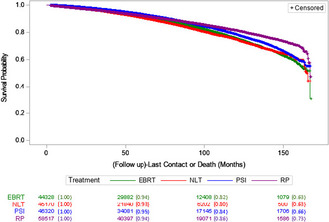

## 1 INTRODUCTION

Patients diagnosed with localized, low‐risk prostate cancer (LRPC)^1^, ^2^ face intricate treatment choices, ranging from active surveillance (AS) to definitive local interventions, including radical prostatectomy (RP), external‐beam radiation therapy (EBRT), or prostate seed implantation (PSI). The selection of the most suitable approach is influenced by various factors, including age, comorbidities, and preferences of both physicians and patients.

In recent years, AS has gained widespread acceptance as a management option for LRPC that transcends demographic boundaries.^3^, ^4^ Studies have demonstrated its safety and feasibility over a 5–10‐year period,^5^, ^6^, ^7^, ^8^ making it the preferred initial management strategy for LRPC.^2^ However, it is essential to note that AS itself does not improve the oncologic outcomes. The long‐term trajectory of AS remains somewhat uncertain, except in cases of very LRPCs.^9^ For example, Klotz et al. reported that the 10‐ and 15‐year cause‐specific survival rates in a single‐arm prospective AS cohort were 98.1% and 94.3%, respectively. Nevertheless, approximately one‐third of the patients initially managed with AS eventually required definitive local treatment.^8^

Indeed, local definitive treatments, RP and radiation, have been shown to reduce disease progression and metastasis in prostate cancer overall (not limited to the LRPC risk group) in prospective clinical trials such as the SPCG4, PIVOT, and ProtecT trials.^10^, ^11^, ^12^ Notably, an increasing number of patients who started observation eventually received local therapies, RP, or radiotherapy over time: 20% within 10 years in the PIVOT trial,^11^ 55% in 10 years, and 61% by 15 years in the ProtecT trial.^12^ Despite these advancements, optimal management of localized LRPC remains a contentious issue. Prospective studies, including the ProtecT trial, often face limitations due to the relatively small sample sizes in each cohort for practical reasons, necessitating extended follow‐up periods spanning decades to discern outcomes.

To address these challenges, we harnessed the extensive patient data from the National Cancer Database (NCDB). Our analysis focused on patients diagnosed with LRPC between 2004 and 2015, with inclusion criteria limited to individuals with Charlson–Deyo comorbidity scores (C/DCS) of 0 or 1 to minimize the potential biases related to comorbidities. Our goal was to enhance our understanding of the overall survival (OS) outcomes associated with various LRPC treatments, thereby assisting in optimizing management strategies.

## 2 MATERIALS AND METHODS

### 2.1 Patient selection

**FIGURE 1:**
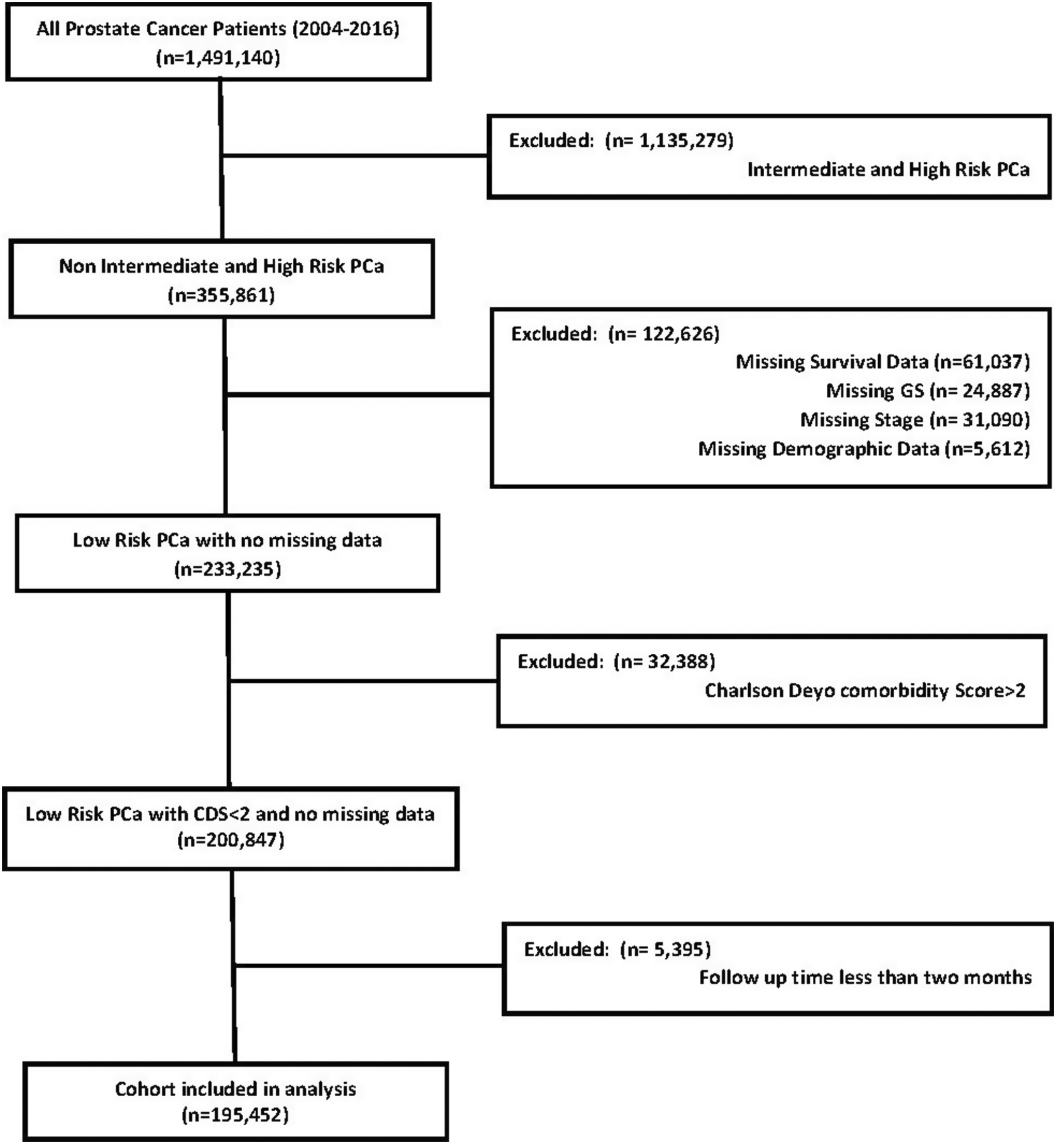
CONSORT diagram.

This retrospective study utilized NCDB data, a comprehensive hospital‐based clinical oncology database supported by the American College of Surgeons' Commission on Cancer (CoC) and the American Cancer Society. The NCDB collects data from over 1500 CoC‐accredited hospitals and covers approximately 70% of newly diagnosed cancer cases in the United States. We focused on patients diagnosed with LRPC between 2004 and 2015, as defined by the D'Amico and NCCN criteria (PSA <10 ng/mL, cT1‐cT2a, and GS ≤6).^1^, ^2^ Patients who did not meet the criteria for LRPC, those with missing clinical staging data, or those with missing survival data were excluded. In NCDB, comorbidities were recorded based on the Charlson–Deyo comorbidity score (C/DCS).^13^ Only patients with a C/DCS score of 0 or 1 were included to maintain cohort comparability. The CONSORT diagram in Figure 1 visually represents the study exclusion criteria, defining the final cohort with 195,452 eligible patients.

The study collected several patient characteristics, including age at diagnosis (years), race (white, black, or other), insurance status (private, Medicaid/other government insurance, Medicare, uninsured), median income (categorized as ≤$30,000, $30,000–35,999, $36,000–45,999, ≥$46,000), and education level (percentage of patients without a high school diploma, categorized as ≥21%, 13–20.9%, 7–12.9%, <7%). The median income and education level were determined using the zip code. The Gleason score, prostate‐specific antigen (PSA) level, TNM stage, and treatment modalities were obtained.

### 2.2 Ethical considerations

No institutional review board approval was required given the use of de‐identified NCDB data, which complies with strict data confidentiality and HIPPA privacy regulations.

### 2.3 Treatment

The primary independent variable of interest was treatment modality: RP, EBRT, PSI, or no local treatment (NLT). Patients who received systemic therapy or other treatment were excluded from the study. Differentiating between AS and other reasons for refusing local treatment was impossible because of NCDB limitations.

### 2.4 Statistical methods

Chi‐square tests were conducted for categorical variables to compare confounder differences among treatment groups, presented as frequency (percentage). Propensity score analysis was employed to address the treatment selection bias resulting from a lack of randomization.^14^ Propensity scores were calculated using multinomial logistic regression based on demographic variables (age, race, insurance status, income, educational level, area, facility type, and race‐facility type interaction). Inverse probability of treatment weight (IPTW) was applied to balance treatment groups.^15^ A chi‐squared test with IPTW as the weight was conducted to validate the balance of the treatment groups.

The primary outcome measure was OS. Kaplan–Meier survival plots stratified by treatment groups were compared using the log‐rank test, and pairwise comparisons were used to determine the significance between treatments.

The accelerated failure time (AFT) model was used to address the violation of the proportional hazard assumption as an alternative to the Cox proportional model.^16^, ^17^, ^18^, ^19^ Because estimating the AFT model's parameters requires assuming a time distribution, the Weibull distribution was chosen as it resulted in the smallest Akaike information criterion and Bayesian information criterion. The AFT models for the treatment groups were stratified by age and other demographics, with all tests adjusted using IPTW.

Statistical analyses were performed using SAS Enterprise Guide 9.4 HF3 with a significance level of *α* = 0.05.

## 3 RESULTS

### 3.1 Patient characteristics

We identified 195,452 LRPC patients in the NCDB database between 2004 and 2015 (Table 1). Most (74%) were under 70 years old, 29% were under 60, and only 10% were aged 75 years or older. The majority were Caucasian (85%), followed by African American (13%) and other races (1.8%). Most LRPC patients (79%) received care in academic programs or comprehensive community cancer programs. Most patients were covered by private (51%) or governmental (46%) insurance, with uninsured patients comprising only 1.3% of the population.

**TABLE 1:** Summary of patient characteristics of overall study population.

Variable	*n* = 195,452
Age	
<60	57,139 (29%)
60–64	42,140 (22%)
65–69	44,968 (23%)
70–74	31,095 (16%)
≥75	20,116 (10%)
Race	
White	191,265 (85%)
AA	28,719 (13%)
Other	4039 (1.8%)
Area	
Metropolitan	160,092 (82%)
Urban	26,937 (14%)
Rural	3477 (1.8%)
Facility type	
Community Cancer Program	15,098 (7.8%)
Comprehensive Community Cancer Program	79,192 (41%)
Academic/Research Program	74,192 (38%)
Integrated Network Cancer Program	26,042 (13%)
Insurance status	
Not insured	2611 (1.3%)
Private	99,523 (51%)
Government	90,172 (46%)
Income*	
<30,000	28,783 (15%)
30,000–34,999	41,441 (21%)
35,000–45,999	51,375 (26%)
46,000+	72,932 (37%)
Education**	
≥21%	28,783 (14%)
13–20.9%	45,310 (23%)
7–12.9%	65,006 (33%)
<7%	57,719 (30%)
Treatment	
NLT	45,462 (23%)
RP	58,501 (30%)
EBRT	44,833 (23%)
PSI	46,656 (24%)
Mortality	24,545 (13%)

Abbreviations: EBRT, external‐beam radiation therapy; NLT, no local treatment; PSI, prostate seed implant; RP, radical prostatectomy.

* Level of income by zipcode.

** Percentage of high school graduates by zipcode.

### 3.2 Treatment modalities

Among the LRPC patients, 23.26% received NLT, 29.93% underwent RP, 22.94% received EBRT, and 23.87% had PSI (Table 1).

In patients receiving local therapies, 99.6% of RP, 99.1% of EBRT, and 99.9% of PSI treatments occurred within the first year of diagnosis, indicating primary rather than deferred therapies.

Table 2 displays patient demographics by treatment modalities, revealing significant differences in age, race, insurance type, income, education, residence, and treatment facility among the NLT, RP, EBRT, and PSI groups. Propensity score analysis was conducted to balance the treatment groups using multinomial logistic regression and IPTW. This achieved group comparability, with no statistically significant differences (*p* > 0.05) in patient demographics between the treatment groups. (Table 2). All further analyses were performed using IPTW‐corrected data.

**TABLE 2:** Chi‐squared test result of demographics and treatments before and after propensity score weighted adjustment.

Variable	NLT (*n* = 45,462)	RP (*n* = 58,501)	EBRT (*n* = 44,833)	PSI (*n* = 46,656)	*p* value
Before propensity score weighted adjustment					
Age					
<60	12,190 (26.81%)	24,863 (42.50%)	8029 (17.91%)	12,055 (25.84%)	<0.0001
60–64	9753 (21.45%)	13,489 (23.06%)	8511 (18.98%)	10,385 (22.26%)	
65–69	10,784 (23.72%)	11,086 (18.95%)	11,526 (25.71%)	11,571 (24.80%)	
70–74	7292 (16.04%)	5260 (8.99%)	10,100 (22.53%)	8442 (18.09%)	
>75	5443 (11.97%)	3803 (6.50%)	6667 (14.87%)	4203 (9.01%)	
Race					
White	38,441 (84.56%)	52,367 (89.51%)	36,747 (81.96%)	39,658 (85.00%)	<0.0001
AA	6055 (13.32%)	5186 (8.86%)	7171 (15.99%)	6242 (13.38%)	
Other	966 (2.12%)	948 (1.62%)	915 (2.04%)	756 (1.62%)	
Insurance					
NI	993 (2.18%)	617 (1.05%)	620 (1.38%)	381 (0.82%)	<0.0001
Private	21,990 (48.37%)	37,306 (63.77%)	17,238 (38.45%)	22,985 (49.26%)	
Gov	21,504 (47.30%)	19,779 (33.81%)	26,180 (58.39%)	22,707 (48.67%)	
Unknown	975 (2.14%)	799 (1.37%)	795 (1.77%)	583 (1.25%)	
Income					
<30,000	6549 (14.47%)	7396 (12.69%)	7627 (17.09%)	7207 (15.54%)	<0.0001
30,000–34,999	9140 (20.20%)	12,090 (20.75%)	9561 (21.42%)	10,650 (22.97%)	
35,000–45,999	11,671 (25.79%)	15,907 (27.30%)	11,641 (26.08)	12,154 (26.21%)	
>46,000	17,890 (39.54%)	22,875 (39.26%)	15,809 (35.42%)	16,358 (35.28%)	
Education					
>21%	6179 (13.64%)	6750 (11.58%)	7241 (16.21%)	6446 (13.89%)	<0.0001
13%–20.9%	9620 (21.24%)	12,947 (22.21%)	11,127 (24.92%)	11,616 (25.03%)	
7%–12.9%	14,696 (32.45%)	19,830 (34.02%)	14,640 (32.78%)	15,838 (34.13%)	
<7%	14,798 (32.67%)	18,770 (32.20%)	11,650 (26.09%)	12,500 (26.94%)	
Area					
Metro	37,416 (82.30%)	47,899 (81.88%)	37,404 (83.43%)	37,369 (80.09%)	<0.0001
Urban	6085 (13.38%)	7805 (13.34%)	5778 (12.89%)	7268 (15.58%)	
Rural	793 (1.74%)	1129 (1.93%)	615 (1.37%)	940 (2.01%)	
Unknown	1168 (2.57%)	1668 (2.85%)	1036 (2.31%)	1079 (2.31%)	
Facility					
CCP	3658 (8.05%)	3766 (6.45%)	4067 (9.07%)	3607 (7.73%)	<0.0001
CCCP	13,161 (28.97%)	23,153 (39.65%)	20,086 (44.81%)	22,787 (48.85%)	
A/RP	23,749 (52.28%)	23,354 (40.00%)	15,086 (33.65%)	12,770 (27.38%)	
INCP	4859 (10.70%)	8115 (13.90%)	5589 (12.47%)	7479 (16.03%)	

After propensity score weighted adjustment					
Age					
<60	13,225 (29.26%)	16,848 (28.79%)	12,682 (28.61%)	13,374 (28.87%)	0.8493
60–64	9698 (21.46%)	12,505 (21.37%)	9534 (21.51%)	9966 (21.51%)	
65–69	10,332 (22.86%)	13,559 (23.17%)	10,324 (23.29%)	10,693 (23.08%)	
70–74	7189 (15.91%)	9469 (16.18%)	7145 (16.12%)	7438 (16.06%)	
>75	4745 (10.50%)	6139 (10.49%)	4648 (10.48%)	4850 (10.47%)	
Race					
W	38,670 (85.57%)	49,743 (85%)	37,817 (85.30%)	39,633 (85.56%)	0.1370
AA	5682 (12.57%)	7672 (13.11%)	5705 (12.87%)	5836 (12.60%)	
Other	838 (1.85%)	1104 (1.89%)	811 (1.83%)	853 (1.84%)	
Insurance					
NI	596 (1.30%)	777 (1.33%)	616 (1.39%)	614 (1.33%)	0.0754
Private	22,890 (50.65%)	29,198 (49.89%)	22,081 (49.81%)	23,294 (50.29%)	
Gov	20,943 (46.35%)	27,657 (47.26%)	20,938 (47.23%)	21,672 (46.79%)	
Unknown	760 (1.68%)	888 (1.52%)	697 (1.57%)	743 (1.60%)	
Income					
<30,000	6659 (14.73%)	8878 (15.17%)	6645 (14.99%)	6878 (14.85%)	0.5011
30,000–34,999	9776 (21.63%)	12,578 (21.49%)	9535 (21.51%)	9985 (21.56%)	
35,000–45,999	12,140 (26.87%)	15,453 (26.41%)	11,740 (26.48%)	12,457 (26.89%)	
>46,000	16,614 (36.77%)	21,610 (36.93%)	16,412 (37.02%)	17,002 (36.70%)	
Education					
>21%	6081 (13.46%)	8205 (14.02%)	6151 (13.87%)	6289 (13.58%)	0.2176
13%–20.9%	10,545 (23.33%)	13,759 (23.51%)	10,405 (23.47%)	10,939 (23.62%)	
7%–12.9%	15,223 (33.69%)	19,446 (33.23%)	14,715 (33.19%)	15,551 (33.57%)	
<7%	13,341 (29.52%)	17,110 (29.24%)	13,062 (29.46%)	13,543 (29.24%)	
Area					
Metro	36,863 (81.57%)	48,056 (82.12%)	36,454 (82.23%)	37,852 (81.71%)	0.3510
Urban	6462 (14.30%)	8108 (13.85%)	6093 (13.74%)	6588 (14.22%)	
Rural	829 (1.83%)	1037 (1.77%)	788 (1.78%)	828 (1.79%)	
Unknown	1036 (2.29%)	1318 (2.25%)	997 (2.25%)	1054 (2.27%)	
Facility					
CCP	3514 (7.78%)	4775 (8.16%)	3564 (8.04%)	3606 (7.78%)	0.1561
CCCP	18,261 (40.41%)	23,935 (40.90%)	18,040 (40.69%)	18,844 (40.68%)	
A/RP	17,390 (38.48%)	22,159 (37.87%)	16,920 (38.17%)	17,708 (38.23%)	
INCP	6024 (13.33%)	7650 (13.07%)	5809 (13.10%)	6165 (13.31%)	

Abbreviations: A/RP, Academic/Research Program; AA, African American; CCCP, Comprehensive Community Cancer Program; CCP, Community Cancer Program; EBRT, external‐beam radiation therapy; Gov, government; INCP, Integrated Network Cancer Program; NI, no insurance; NLT, no local treatment, RP, radical prostatectomy; PSI, prostate seed implant.

### 3.3 Survival outcomes

**FIGURE 2:**
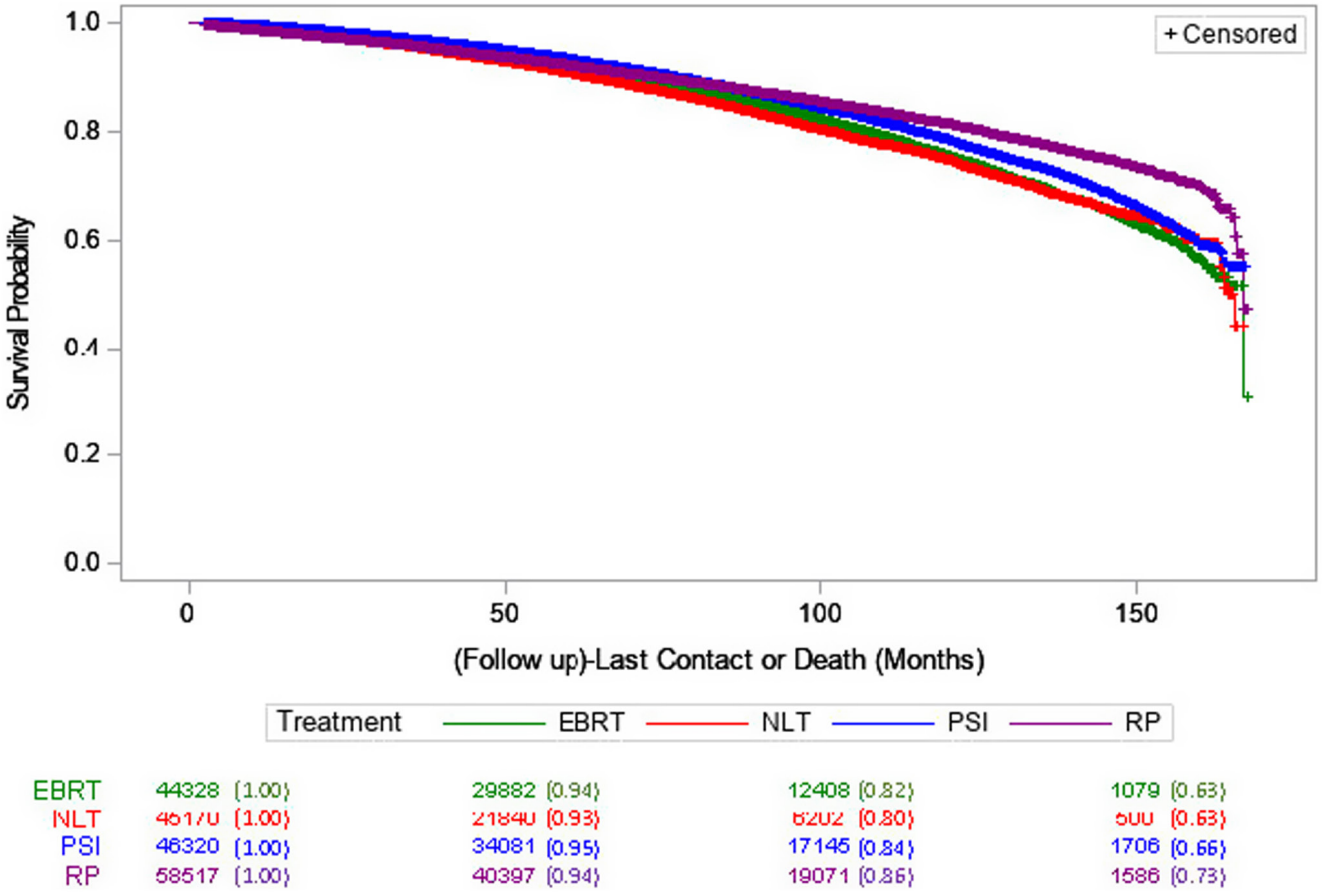
Kaplan–Meier plot for overall survival probabilities based on treatment modalities.

During a median follow‐up of 70.8 months, 24,545 deaths occurred, resulting in an all‐cause mortality rate of 13%. Figure 2 presents the Kaplan–Meier plot depicting OS probabilities following IPTW adjustment for different management modalities. In the initial 50 months, the survival probabilities for the four treatment modalities overlapped, signifying comparable survival among the NLT, surgery, and radiation options. Significant differences in survival rates emerged after 75 months, with RP consistently maintaining the highest survival rate, whereas NLT exhibited the lowest survival rate among the treatment groups beyond 75 months (Figure 2 and Table S2). The Kaplan–Meier plot without IPTW adjustment is shown in Figure S1. Log‐rank test revealed significant differences in survival probabilities between treatments (Table S3). Survival probabilities estimated by AFT model differed significantly among the treatment groups (*p* < 0.0001) with the odds ratios of 1.21, 1.05, and 1.13 for RP, EBRT, and PSI, respectively, compared to NLT (Table 3), indicating improvement of survival for RP, EBRT, and PSI over NLT.

**TABLE 3:** Accelerated failure time model for overall survival rates among treatment groups compared to NLT.

Treatments	Estimate	Odds ratio	S.E.	Chi‐square	*p* value
NLT	Reference	–	–	–	–
RP	0.19	1.21	0.01	233.21	<0.0001
EBRT	0.05	1.05	0.01	14.97	0.0001
PSI	0.12	1.13	0.01	96.23	<0.0001

Abbreviations: EBRT, external‐beam radiation therapy; NLT, no local treatment; PSI, prostate seed implant; RP, radical prostatectomy; S.E., standard error.

### 3.4 Survival by age groups

Along with comorbidities, age significantly influences treatment decisions in clinical practice. Table 4 presents a comparison of the survival rates of the different treatment modalities relative to NLT across various age groups. RP significantly improved survival by 47% (1.47, *p* < 0.0001), 34% (1.34, *p* < 0.0001), 27% (1.27, *p* < 0.0001), and 10% (1.10, *p* = 0.0002) in the age groups <60, 60–64, 65–69, and 70–74, respectively. However, the benefit of RP decreased with advancing age, leading to an 11% decrease in survival (0.89, *p* < 0.0001) in men aged >75 years. In contrast, EBRT did not improve survival in younger age groups; rather, it decreased survival by 11% (0.89, *p* = 0.0020), and 9% (0.91, *p* = 0.0111) in patients aged <60, and 60–64, respectively. However, EBRT significantly improved survival in older patients by 6% (1.06, *p* = 0.0160) and 27% (1.27, *p* < 0.0001) in those aged 70–74 and over 75 years, respectively. Similarly, PSI did not improve survival in age groups younger than 64 years but exhibited significant survival benefits of 6% (1.06, *p* = 0.0242), 11% (1.11, *p* < 0.0001), and 34% (1.34, *p* < 0.0001) compared to NLT in age groups of 65–69, 70–74, and over 75, respectively. Thus, the survival advantages of EBRT and PSI increased with age, particularly in patients older than 70 years for EBRT and 65 years for PSI. In essence, NLT yielded non‐inferior survival outcomes for younger patients compared with radiation modalities when surgery was not chosen.

**TABLE 4:** Accelerated failure time model results for each treatment stratified by age.

Treatment	<60		60–64		65–69		70–74		>75	
	OR	*p* value	OR	*p* value	OR	*p* value	OR	*p* value	OR	*p* value
NLT	Reference	–	–	–	–	–	–	–	–	–
RP	1.47	<0.0001	1.34	<0.0001	1.27	<0.0001	1.10	0.0002	0.89	<0.0001
EBRT	0.89	0.0020	0.91	0.0111	0.99	0.7234	1.06	0.0160	1.27	<0.0001
PSI	0.97	0.4307	1.01	0.7933	1.06	0.0242	1.11	<0.0001	1.34	<0.0001

Abbreviations: EBRT, external‐beam radiation therapy; NLT, no local treatment; OR, odds ratio; PSI, prostate seed implant; RP, radical prostatectomy.

### 3.5 Survival by race and insurance

RP, EBRT, and PSI consistently demonstrated significant survival benefits across racial groups, mirroring trends in most demographics except for the uninsured or other racial groups (Table S4). EBRT and PSI predominantly benefited those with government insurance and lower‐income brackets but not those with private insurance or no insurance, potentially reflecting the patient population with Medicare coverage, that is, older age groups, aligning with the data shown in Table 4.

### 3.6 Survival by income and education

RP improved survival regardless of income and education level, whereas EBRT and PSI primarily benefited lower‐income patients and potentially older individuals. Both EBRT and PSI were advantageous at varying educational levels, except for those with lower education levels (<7%), suggesting a potential disparity (Table S4).

### 3.7 Survival by location and facility

EBRT benefited patients in metropolitan areas, whereas PSI showed survival benefits across all locations, suggesting the role of treatment logistics in radiation techniques. Treatment benefits varied among the different facilities for both EBRT and PSI (Table S4).

## 4 DISCUSSION

In this population‐based retrospective study of LRPC, we analyzed various treatment outcomes using NCDB data, encompassing nearly 200,000 patients, which is the largest LRPC‐focused study to date. Our findings revealed distinct survival outcomes among different treatment modalities. RP exhibited lower all‐cause mortality, a benefit observed across demographics up to the age of 74 years, but diminishes with age. The survival of patients with NLT was comparable to that of patients with EBRT and PSI, except for older patients. EBRT and PSI confer survival benefits mainly for those over 65 years (PSI) and 70 years (EBRT).

The survival benefit of RP has been established historically,^10^, ^20^, ^21^, ^22^ primarily for intermediate‐risk prostate cancer. However, this study, for the first time, unveiled the advantage of RP in LRPC owing to its substantial sample size of 50,000 patients per group. It addresses the limitation of small sample sizes in prospective studies and enhances the statistical power for treatment difference detection.

The long‐term survival advantage of RP over NLT in LRPC is conceivable because NLT alone lacks a direct impact on survival. Despite its low‐risk nature, LRPC can progress and develop metastasis over time. An extended follow‐up study revealed a 15‐year prostate cancer‐specific mortality rate of 5.7% for AS.^8^ Without deferred local intervention, mortality could increase to 11% (15 years) and 25% (20 years), respectively.^23^ It is worth noting that most patients in this study received primary local treatment within the first year of diagnosis. Only a tiny fraction of patients (<0.4% for RP, <0.9% for EBRT, <0.1% for PSI) received local treatment longer than a year later from the time of diagnosis. In a recent Cochrane systematic review,^24^ RP was found to improve oncologic outcomes compared to watchful waiting (WW) and probably reduced the risks of disease progression and metastatic disease, but similar survival outcomes, with limited 10‐year follow‐up, compared to AS.

This study found that RP outperformed EBRT and PSI in terms of LRPC survival. We hypothesized that radiation may not fully eradicate prostate tissue, potentially leaving behind surviving tissue that fosters synchronous cancer development due to multifocality and field cancerization from ongoing environmental carcinogen exposure or genetic predisposition. Prostate cancer stem cells may also contribute to therapy resistance.^25^ Therefore, the therapeutic effects of radiation may not be durable, which could explain the negative impact of EBRT on survival in younger patients. The ability of RP to eradicate prostate tissue and cancer stem cells reduces the risk of synchronous or secondary prostate cancer. Furthermore, because of the inherent risk of sampling bias with prostate biopsy, higher‐risk prostate cancer with occult, synchronous, or metachronous high‐grade disease missed by biopsy could be included in the radiation groups.

The benefit of RP was shown across racial groups between black and white, insurance types, income levels, neighborhoods of various education levels, regions of living across metro, urban, and rural, and facility types, except for those of other races, uninsured, and of the unknown area of living. This latter population also did not benefit from EBRT and PSI, likely reflecting disparities and potentially increased confounding risks associated with underlying social and economic factors. Radiation seemed beneficial across race and education levels, particularly to those with government insurance and with relatively lower incomes, consistent with the observation from the age‐controlled analysis (Table 4) that radiation benefits more to those of older age groups, typically the Medicare population. In addition, the difference of treatment logistics between EBRT and PSI, accessibility to facilities, and social support, including transportation, could also impact the choices and benefits of radiation (Table S4).

In recent years, the management of LRPC has undergone a significant paradigm shift, with AS rapidly becoming the dominant choice since 2010^3^ because of societal recommendations. Current guidelines from AUA/ASTRO/SUO and NCCN endorse AS as a safe approach for LRPC, especially in patients with very low‐risk prostate cancer.^2^, ^26^ While AS may not directly improve oncologic outcomes in LRPC, it offers the advantage of preserving the quality of life by avoiding treatment‐related side effects. This is particularly important, given the lack of a universally recognized optimal treatment for LRPC. Our study highlights that apart from RP, NLT demonstrated non‐inferior survival outcomes to EBRT or PSI in younger age groups. Thus, if RP is not the course of action chosen by patients and providers, NLT represents a reasonable alternative for individuals under the age of 65.

The findings of this study are hypothesis‐generating. The multifocal, heterogeneous, and dynamic nature of prostate cancer with potential low‐ to high‐grade progression^27^, ^28^ creates uncertainties in clinical management. The clinical criteria we base our treatment decisions upon may be inadequate and imprecise.^29^ To address these concerns, the National Institute for Health and Clinical Excellence recommends the use of multiparametric MRI as a prerequisite for AS. Additionally, repeat prostatic biopsy during AS aids in tracking disease progression and changes over time. The current AUA/ASTRO/SUO guidelines advocate RP or radiotherapy as suitable for LRPC patients with risk factors, including perineural invasion, African American race, family history, or genetic predisposition to lethal or metastatic prostate cancer.^26^

The strength of our study lies in its unprecedented large sample size, focusing on LRPC, bolstering both reliability and statistical power. The even distribution among the various treatment groups, inclusion of patients with low C/DCS, and meticulous age‐controlled analysis ensured robust comparability. Furthermore, this study encompassed a substantial representation of black patients (13%), offering valuable insights into the potential disparities. However, we must acknowledge the inherent limitations of our NCDB‐based investigation, notably its retrospective and non‐randomized nature, which may have introduced selection bias despite rigorous efforts to control for age and comorbidities, even with meticulous endeavors using propensity score analysis to balance different cohorts. It is also worth noting that the inability to distinguish between AS and WW in the NLT category owing to the nature of NCDB data limits our ability to assess the specific impact of each approach. While NLT in younger patients treated at academic institutions likely represents AS and most likely WW in those older than 75 years, determining the intention of NLT for patients between 70 and 74 years becomes more challenging due to the limited information available in the NCDB dataset. The Gleason scoring modification in 2005 led to risk group migration in some previously thought to be low Gleason scores upgraded to scores of 7 or higher.^30^, ^31^ Given that our patient population spanned from 2004 to 2015, it is conceivable that some patients from before 2005 may have been classified as having Gleason 7 under the modified Gleason system, making it challenging to fully assess the implications of these changes in our study's conclusion. Furthermore, because of the inherent risk of sampling bias with prostate biopsy and the use of non‐MRI‐targeted biopsies, higher‐risk prostate cancer with occult, synchronous, or metachronous, high‐grade disease missed by biopsy could be included in the radiation groups or the NLT group, especially when the data are primarily from the pre‐MRI era. Lastly, because of the nature of NCDB data, information regarding patients' symptoms, including urinary obstruction, previous sexual function, prostate volume, quality of life, and disease‐specific mortality, may not be obtained, which could play a role in treatment decisions and outcomes.

## 5 CONCLUSION

Our study highlights the differences in survival among treatments for LRPC. RP confers a survival advantage to younger LRPC patients, diminishing with age and being absent in those over 75 years of age. Conversely, EBRT and PSI benefit older patients, specifically those over 70 years of age for EBRT and 65 years of age for PSI. Notably, NLT demonstrated non‐inferior survival to EBRT and PSI in younger patients, thus presenting a reasonable alternative for those opting against RP.

## AUTHOR CONTRIBUTIONS

**Shifeng Mao:** Conceptualization (lead); data curation (lead); formal analysis (lead); investigation (lead); methodology (lead); project administration (lead); resources (lead); supervision (lead); validation (lead); visualization (lead); writing – original draft (lead); writing – review and editing (lead). **Arash Samiei:** Conceptualization (equal); formal analysis (equal); methodology (equal); writing – original draft (equal); writing – review and editing (equal). **Yue Yin:** Data curation (lead); formal analysis (equal); methodology (equal); validation (lead); writing – original draft (equal); writing – review and editing (equal). **Rodney E. Wegner:** Formal analysis (equal); methodology (equal); writing – original draft (equal); writing – review and editing (equal). **Angela Sanguino:** Methodology (equal); writing – original draft (equal); writing – review and editing (equal). **John Lyne:** Writing – original draft (equal); writing – review and editing (equal). **Ralph Miller:** Writing – original draft (equal); writing – review and editing (equal). **Jeffrey Cohen:** Conceptualization (equal); supervision (equal); writing – original draft (equal); writing – review and editing (equal).

## FUNDING INFORMATION

No funding was received for this study.

## CONFLICT OF INTEREST STATEMENT

Shifeng Mao received honorarium payments for consultancy from Sanofi, AstraZeneca, Pfizer, SeaGen, Astellas, Bayer, Exelixis, and Cardinal Health for consultancy and for speaker bureau from Bristol‐Myers Squibb in the past. However, none of the consultancy and speaker bureau activities were relevant to the content of this manuscript. The authors have no conflicts of interest to declare.

### ETHICS STATEMENT

This retrospective review was performed using de‐identified patient data. Given patient de‐identification, the study was exempt from institutional review board supervision.

## Supporting information


Figure S1.

Table S2.

Table S3.

Table S4.

## Data Availability

DATA Availability StatementData supporting the study's findings are available from the National Cancer Database (NCDB). Restrictions apply to the availability of these data, which were used under the license for this study. Data are available from the corresponding author with permission from the NCDB.
