# Supplementary figures and images for: The survival outcomes of localized low‐risk prostate cancer, a population‐based study using NCDB
Source: Cancer Med. 2024 Aug 9;13(15):e70060. doi: 10.1002/cam4.70060 (PMC11310764; doi:10.1002/cam4.70060)

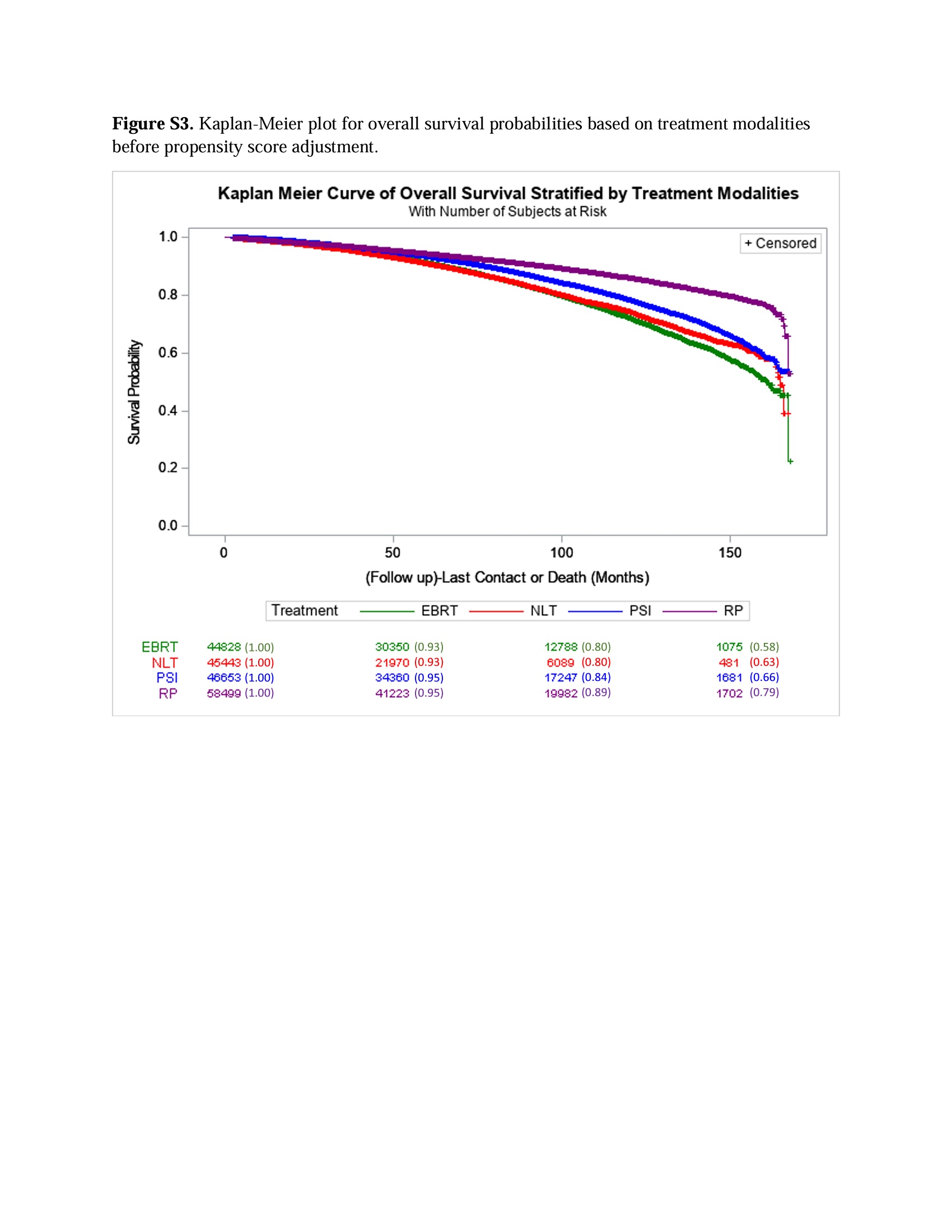

Supplement: Supplementary file 1 — Figure S1. [file CAM4-13-e70060-s002.jpeg]
