# Supplementary material for: The survival outcomes of localized low‐risk prostate cancer, a population‐based study using NCDB
Source: Cancer Med. 2024 Aug 9;13(15):e70060. doi: 10.1002/cam4.70060 (PMC11310764; doi:10.1002/cam4.70060)
Supplement: Supplementary file 2 — Table S2. [file CAM4-13-e70060-s001.docx]

**Table S2**. Survival rate based on treatment modality

|  | 50 months | 75 months | 100 months | 125 months | 150 months |
| --- | --- | --- | --- | --- | --- |
| NLT | 0.93 | 0.87 | 0.80 | 0.73 | 0.63 |
| RP | 0.94 | 0.90 | 0.86 | 0.80 | 0.73 |
| EBRT | 0.94 | 0.89 | 0.82 | 0.74 | 0.63 |
| PSI | 0.95 | 0.91 | 0.84 | 0.77 | 0.66 |

*Note.* NLT: no local treatment; RP: radical prostatectomy; EBRT: external-beam radiation therapy; PSI: prostate seed implant.
