# Supplementary material for: The survival outcomes of localized low‐risk prostate cancer, a population‐based study using NCDB
Source: Cancer Med. 2024 Aug 9;13(15):e70060. doi: 10.1002/cam4.70060 (PMC11310764; doi:10.1002/cam4.70060)
Supplement: Supplementary file 3 — Table S3. [file CAM4-13-e70060-s003.docx]

**Table S3**. Log-rank test of survival probabilities between treatments

| Test | Treatment | Chi-square | DF | P value |
| --- | --- | --- | --- | --- |
| Overall | All | 291.48 | 3 | <.0001 |
| Pairwise comparison | NLT vs RP | 207.21 | 1 | <.0001 |
|  | NLT vs EBRT | 23.8 | 1 | <.0001 |
|  | NLT vs PSI | 138.04 | 1 | <.0001 |
|  | RP vs EBRT | 140.96 | 1 | <.0001 |
|  | RP vs PSI | 30.70 | 1 | <.0001 |
|  | EBRT vs PSI | 51.88 | 1 | <.0001 |

*Note*. DF: degree of freedom; NLT: no local treatment; RP: radical prostatectomy; EBRT: beam radiation therapy; PSI: prostate seed implant.
