# Supplementary material for: The survival outcomes of localized low‐risk prostate cancer, a population‐based study using NCDB
Source: Cancer Med. 2024 Aug 9;13(15):e70060. doi: 10.1002/cam4.70060 (PMC11310764; doi:10.1002/cam4.70060)
Supplement: Supplementary file 4 — Table S4. [file CAM4-13-e70060-s004.docx]

**Table S4**: Treatment modalities stratified by demographics

| **Race** | | | | | | |
| --- | --- | --- | --- | --- | --- | --- |
| Treatment | White | | Black | | Other | |
|  | OR | P value | OR | P value | OR | P value |
| NLT | Reference | - | - | - | - | - |
| RP | 1.20 | <.0001 | 1.33 | <.0001 | 1.11 | .3147 |
| EBRT | 1.03 | .0216 | 1.20 | <.0001 | 0.94 | .5435 |
| PSI | 1.10 | <.0001 | 1.35 | <.0001 | 1.10 | .3784 |

| **Insurance** | | | | | | | | |
| --- | --- | --- | --- | --- | --- | --- | --- | --- |
| Treatment | No insurance | | Private | | Government | | Unknown | |
|  | OR | P value | OR | P value | OR | P value | OR | P value |
| NLT | Reference | - | - | - | - | - | - | - |
| RP | 1.24 | .1151 | 1.28 | <.0001 | 1.16 | <.0001 | 1.42 | .0006 |
| EBRT | 1.11 | .4563 | 0.90 | <.0001 | 1.14 | <.0001 | 1.01 | .9528 |
| PSI | 0.92 | .5160 | 0.97 | .2733 | 1.22 | <.0001 | 1.07 | .5126 |

| **Income** | | | | | | | | |
| --- | --- | --- | --- | --- | --- | --- | --- | --- |
| Treatment | < 30000 | | 30000-34999 | | 35000-45999 | | >46000 | |
|  | OR | P value | OR | P value | OR | P value | OR | P value |
| NLT | Reference | - | - | - | - | - | - | - |
| RP | 1.27 | <.0001 | 1.26 | <.0001 | 1.17 | <.0001 | 1.17 | <.0001 |
| EBRT | 1.14 | <.0001 | 1.11 | <.0001 | 1.00 | .8441 | 0.99 | .7120 |
| PSI | 1.27 | <.0001 | 1.21 | <.0001 | 1.11 | <.0001 | 1.02 | .5189 |

| **Education** | | | | | | | | |
| --- | --- | --- | --- | --- | --- | --- | --- | --- |
| Treatment | >21% | | 13%-20.9% | | 7%-12.9% | | <7% | |
|  | OR | P value | OR | P value | OR | P value | OR | P value |
| NLT | Reference | - | - | - | - | - | - | - |
| RP | 1.18 | <.0001 | 1.25 | <.0001 | 1.23 | <.0001 | 1.16 | <.0001 |
| EBRT | 1.11 | .0019 | 1.06 | .0254 | 1.05 | .0150 | 1.00 | .9414 |
| PSI | 1.18 | <.0001 | 1.16 | <.0001 | 1.17 | <.0001 | 1.02 | .3759 |

| **Area** | | | | | | | | |
| --- | --- | --- | --- | --- | --- | --- | --- | --- |
| Treatment | Metro | | Urban | | Rural | | Unknown | |
|  | OR | P value | OR | P value | OR | P value | OR | P value |
| NLT | Reference | - | - | - | - | - | - | - |
| RP | 1.21 | <.0001 | 1.21 | <.0001 | 1.40 | <.0001 | 1.10 | .2830 |
| EBRT | 1.05 | .0004 | 1.03 | .3189 | 1.09 | .2687 | 1.03 | .7481 |
| PSI | 1.12 | <.0001 | 1.18 | <.0001 | 1.20 | .0241 | 1.05 | .5711 |

| **Facility type** | | | | | | | | |
| --- | --- | --- | --- | --- | --- | --- | --- | --- |
| Treatment | CCP | | CCCP | | A/RP | | INCP | |
|  | OR | P value | OR | P value | OR | P value | OR | P value |
| NLT | Reference | - | - | - | - | - | - | - |
| RP | 1.14 | .0009 | 1.26 | <.0001 | 1.17 | <.0001 | 1.20 | <.0001 |
| EBRT | 1.17 | .0003 | 1.11 | <.0001 | 0.97 | .2338 | 0.96 | .2467 |
| PSI | 1.25 | <.0001 | 1.24 | <.0001 | 0.98 | .3862 | 1.08 | .0255 |

Note. CCP: Community Cancer Program; CCCP: Comprehensive Community Cancer Program; A/RP: Academic/Research Program; INCP: Integrated Network Cancer Program; NLT: no local treatment; RP: radical prostatectomy; EBRT: external-beam radiation therapy; PSI: prostate seed implant.
